# The effect of STI screening during pregnancy on vertical transmission of HIV and adverse pregnancy outcomes in South Africa: a modelling study

**DOI:** 10.1002/jia2.26410

**Published:** 2025-01-26

**Authors:** Dorothy C. Nyemba, Dvora L. Joseph‐Davey, Sinead Delany‐Moretlwe, Landon Myer, Leigh F. Johnson

**Affiliations:** ^1^ Division of Epidemiology and Biostatistics School of Public Health University of Cape Town Cape Town South Africa; ^2^ Wits RHI University of the Witwatersrand Johannesburg South Africa; ^3^ Division of Infectious Diseases Geffen School of Medicine University of California Los Angeles Los Angeles California USA; ^4^ Centre for Integrated Data and Epidemiological Research School of Public Health University of Cape Town Cape Town South Africa

**Keywords:** STI screening, vertical HIV transmission, *Chlamydia trachomatis*, *Trichomonas vaginalis*, *Neisseria gonorrhoeae*, South Africa

## Abstract

**Introduction:**

Sexually transmitted infections (STIs) in pregnancy are associated with an increased risk of vertical HIV transmission and adverse pregnancy and birth outcomes. In South Africa, syndromic management is the standard of care for STI management. We assessed the potential impact of point‐of‐care (POC) screening for curable STIs (*Chlamydia trachomatis* [CT], *Trichomonas vaginalis* [TV] and *Neisseria gonorrhoeae* [NG]) during pregnancy on vertical HIV transmission and adverse pregnancy and birth outcomes.

**Method:**

We developed a static mathematical model to estimate the impact of syndromic management compared to POC screening of STIs in pregnant women attending antenatal clinics in South Africa over one calendar year (2022). Our model assumptions regarding the effect of CT, NG and TV on adverse pregnancy/birth outcomes and vertical HIV transmission were informed by two separate meta‐analyses that we conducted. Local studies informed estimates of STI prevalence, POC screening uptake and treatment, and sensitivity of syndromic management.

**Results:**

In the absence of POC screening for curable STIs, 25.5% of pregnant women without HIV and 34.6% of pregnant women living with HIV were estimated to have undiagnosed and untreated STIs. In the POC scenario, 92% (95% CI: 85−100%) of STIs were diagnosed and treated during pregnancy, reducing antenatal maternal HIV incidence by 10.0% (95% CI: 1.0−20.1%). Overall, vertical HIV transmission was anticipated to reduce by 8.6% (5.2−13.8%), with reductions of 20.9% (15.2−27.0%) at birth and 2.5% (−0.9% to 9.0%) postnatally, in the POC screening scenario compared to current syndromic management. POC screening of curable STIs is further estimated to reduce the incidence of stillbirth by 10.1% (1.3–18.7%), preterm delivery by 6.3% (3.4–9.7%), infants born small for gestational age by 2.7% (0.7–4.9%) and low birth weight by 9.1% (0.9–18%).

**Conclusions:**

POC STI screening and treatment may modestly reduce maternal HIV incidence, vertical HIV transmission, and the risk of adverse pregnancy and birth outcomes, and would substantially reduce the burden of curable STIs in pregnancy. The study provides evidence to move beyond the syndromic management of STIs in South Africa, particularly in antenatal care.

## 1 INTRODUCTION

Sexually transmitted infections (STIs) are among the most common communicable diseases worldwide. The World Health Organization (WHO) estimated that there were 374 million incident cases of the four most common curable STIs, namely *Chlamydia trachomatis* (CT), *Trichomonas vaginalis* (TV), *Neisseria gonorrhoeae* (NG) and syphilis globally in 2020 [1]. Incidence varies widely by region [2−4], with the African region experiencing the highest burden [5]. In most African countries, syndromic management is used for STI care, which involves treating symptomatic infections based on observed signs and symptoms [4, 6]. However, the effectiveness of syndromic management at the population level is limited because of the undertreatment of asymptomatic STIs, and overtreatment of people (especially women) with genital symptoms not associated with an STI. This contributes to antibiotic resistance and leads to unnecessary costs [6]. Point‐of‐care (POC) screening for curable STIs utilizes rapid diagnostic testing at the site of patient care. This approach eliminates laboratory delays, facilitating timely results that enable same‐day treatment and reduce the risk of complications and ongoing transmission [7]. Importantly, POC screening identifies asymptomatic infections that are frequently missed with syndromic management and minimizes overtreatment by avoiding false positives, ensuring treatment is provided only to those with confirmed infections. Improved diagnostic approaches are crucial for addressing challenges posed by syndromic management and enhancing STI management worldwide. Curable STIs are common among pregnant women in sub‐Saharan Africa, with prevalence ranging from 3.3% for NG to 13.8% for TV [8]. South Africa has some of the highest STI prevalence levels among pregnant women [9−12], with most of the infections being asymptomatic. Consistent with WHO guidelines, South Africa currently uses syndromic management to screen for and treat curable STIs, including in pregnant women [13].

The impact of STIs on the sexual transmission of HIV has been extensively researched. Genital tract infections are associated with elevated HIV‐1 shedding [14], and this in turn increases the risk of HIV transmission [11]. STIs that cause mucosal inflammation and ulcers contribute to increased HIV infectivity and susceptibility [15, 16]. Therefore, pregnant women with STIs may be at increased biological risk for HIV acquisition [17]. In pregnant women living with HIV (WLHIV), studies demonstrate that coinfection with curable STIs increases the risk of vertical transmission of HIV [18−22]. For example, one study showed that WLHIV who had STI co‐infection, especially CT and NG, had a 3.5 times greater risk of vertical transmission of HIV to their infants relative to WLHIV without STI co‐infection [19]. However, the effect of STIs on vertical HIV transmission remains less established than the effect of STIs on sexual transmission of HIV, and the evidence has not been systematically reviewed.

In 2019, South Africa had a high antenatal HIV prevalence of 30%, which had remained unchanged since 2004 [23, 24]. However, the most recent antenatal HIV sentinel survey in 2022 indicates a decrease in prevalence to 27.5% at the national level [25]. Despite successful vertical HIV transmission prevention programmes reducing paediatric HIV prevalence to approximately 1% [26], the success of these programmes is compromised by newly acquired maternal HIV during pregnancy or lactation [20−22]. Women with curable STIs face an elevated risk of acquiring HIV during pregnancy [14, 15], and many STIs remain undiagnosed and untreated, contributing to a heightened risk of vertical HIV transmission during pregnancy, childbirth and breastfeeding. To reduce vertical HIV transmission, strategies to enhance STI case management during pregnancy need to be improved, such as including POC screening and treatment of curable STIs in antenatal care (ANC).

Curable STIs in pregnancy have also been associated with several adverse pregnancy and birth outcomes. CT infections during pregnancy have been associated with stillbirth [27, 28], preterm birth, low birthweight (LBW) and small for gestational age (SGA) [29]. Vertical transmission of CT can lead to infant conjunctivitis and pneumonia [19, 30, 31]. Maternal NG is associated with preterm birth, LBW and SGA [32, 33]. Maternal TV infection induces inflammatory responses which may facilitate preterm birth and LBW [34]. It is worth noting that preterm birth remains one of the leading contributors to neonatal deaths [35, 36], and infants born with LBW or SGA are at elevated risk of poor health outcomes [37]. The double burden of STIs and HIV in pregnant women poses a major threat to maternal and child health.

Treatment of maternal curable STIs may reduce these adverse pregnancy and birth outcomes. A randomized trial of presumptive STI therapy during pregnancy in Uganda showed that STI treatment reduced LBW and preterm delivery (PTD) [38]. However, the WANTAIM trial, focusing on POC testing and treatment for CT, NG, TV and bacterial vaginosis during pregnancy, did not result in a decrease in preterm birth and LBW overall. Nevertheless, in a pre‐specified sub‐group analysis, women with NG had a 53% (95% confidence interval: 12−75%) lower risk of preterm birth and LBW [39, 40]. The latest findings from the Madou study in Botswana, which evaluated the diagnosis and treatment of CT and NG, showed a reduced prevalence of preterm birth and LBW in the group that received POC screening and treatment compared to standard of care group (11% vs. 16%), but this difference was not statistically significant [41, 42]. Another randomized controlled trial to evaluate the effect of aetiological screening and treatment of CT, TV and NG on adverse pregnancy and birth outcomes is currently being conducted in South Africa [43]. There is thus mixed evidence regarding the benefits of diagnostic screening and treatment of curable STIs during pregnancy in reducing adverse pregnancy and birth outcomes. A mathematical model has the capacity to integrate inconsistent data from various sources to simulate the effects of introducing POC screening and treatment for curable STIs during pregnancy, in comparison to syndromic management. This approach provides a more comprehensive understanding that can guide and inform public health interventions.

To predict the potential impact of different strategies for screening and treating CT, TV and NG, we developed a model comparing POC screening and treating curable STIs with syndromic management, in pregnant women in South Africa.

## 2 METHODS

We developed a static mathematical model to estimate the impact of syndromic management compared to POC screening and treatment of curable STIs (CT, TV and NG) in pregnant women attending antenatal clinics in South Africa over one calendar year (2022). The model illustrates the effects of including POC screening and treatment of curable STIs in pregnant women attending ANC at a specific point in time, without accounting for any temporal changes. Table 1 presents the model parameters, which were drawn from the literature, or from other models. We performed a rigorous selection of parameters for the model by considering peer‐reviewed studies to ensure reliable estimates. In instances where recent data from South Africa was unavailable, we drew upon earlier studies (recognizing that STI prevalence rates and adverse pregnancy rates have been stable in recent decades [44]) or research from other countries as the next best alternatives.

**Table 1:** Model parameter values

Parameter	Units	Value	Range	Source
Prevalence of curable STIs in WLHIV at first ANC^a^	Proportion	38%	(29−49%)	Davey et al. [9]
Prevalence of curable STIs in women without HIV at first ANC^a^	Proportion	28%	(20−36%)	Davey et al. [9]
Proportion correctly treated in syndromic scenario	Proportion	9%	(3−17%)	Davey et al. [9]
Proportion treated in POC testing scenario	Proportion	92%	(85−100%)	De Voux et al. [45]
Effect of STIs on vertical transmission at/before birth	Relative risk	1.75	(1.54−1.99)	Meta‐analysis (Figure S1)
Effect of STIs on maternal HIV incidence	Relative risk	1.50	(1.00−2.00)	Johnson et al. [48]
Prevalence of stillbirth	Proportion	2.00%	(1.50−2.50%)	DHIS: for 2018–2020, Maswime et al. [49]
Prevalence of preterm deliveries (PTDs)	Proportion	12.5%	(11.4−13.7%)	Ramokolo et al. [50]
Prevalence of small for gestational age (SGA)	Proportion	14.9%	(13.8−16.1%)	Ramokolo et al. [50]
Prevalence of low birth weight (LBW)	Proportion	10.7%	(10.0−11.5%)	Ramokolo et al. [50]
Effect of curable STIs on stillbirth	Odds ratio	1.46	(1.09−1.97)	Meta‐analysis (Table S2)
Effect of curable STIs on PTD	Odds ratio	1.32	(1.18−1.48)	Meta‐analysis (Table S2)
Effect of curable STIs on SGA	Odds ratio	1.14	(1.05−1.25)	Meta‐analysis (Table S2)
Effect of curable STIs on LBW	Odds ratio	1.48	(1.06−2.06)	Meta‐analysis (Table S2)
**Parameter adopted from the Thembisa 4.5 model**				
Annual number of births to WLHIV in 2022	Absolute number	269,832		Thembisa 4.5 estimate
Annual number of WLHIV on ART at conception, in 2022	Absolute number	198,647		Thembisa 4.5 estimate
Total live births in 2022	Absolute number	1,126,490		Thembisa 4.5 estimate
Annual HIV incidence rate in pregnant and breastfeeding women, status quo scenario	Rate	0.01067		Thembisa 4.5 estimate
Average vertical transmission at birth in untreated WLHIV	Proportion	0.167		Match Thembisa output
Average vertical transmission at birth in acutely infected WLHIV	Proportion	0.254		Thembisa 4.5 estimate
Average vertical transmission in WLHIV on ART before conception	Proportion	0.003		Thembisa assumption
Average vertical transmission rate in WLHIV starting ART during pregnancy	Proportion	0.01		Match Thembisa assumption
Average gestation (weeks) at first ANC visit	Absolute number	23		Thembisa assumption
Average gestation (weeks) at ANC retesting visit	Absolute number	34		Thembisa assumption
Average gestation (weeks) at delivery	Absolute number	39		Thembisa assumption
Window period on rapid test (weeks)	Absolute number	4		Thembisa assumption
Sensitivity of rapid testing (excluding acute infection)	Proportion	97.5%		Thembisa assumption
% of untreated WLHIV tested at first antenatal visit	Proportion	98.0%		Thembisa assumption
Probability of being tested later in pregnancy if missing first ANC visit test	Probability	0.475		Thembisa assumption
Probability of being retested later in pregnancy if tested at first ANC visit	Probability	0.76		Thembisa assumption
% of newly diagnosed WLHIV who start ART during pregnancy	Proportion	95.0%		Thembisa assumption
Postnatal transmission probability: Untreated WLHIV	Probability	0.186		Match Thembisa output
Postnatal transmission probability: Untreated acutely infected WLHIV	Probability	0.27		Johnson et al. [51]
Postnatal transmission probability: WLHIV who started ART antenatally	Probability	0.0102		Match Thembisa assumption
Postnatal transmission probability: WLHIV on ART prior to conception	Probability	0.0018		Match Thembisa assumption
% reduction in STI prevalence while breastfeeding, due to POC ANC screening	Proportion	50%	(0−100%)	No data
Average duration of breastfeeding (months) in women without HIV	Absolute number	15.6		Thembisa assumption

Abbreviations: ANC, antenatal clinic; ART, antiretroviral therapy; WLHIV, women living with HIV.

^a^ Curable STIs refer to *Chlamydia trachomatis, Neisseria gonorrhoeae* and *Trichomonas vaginalis*.

### 2.1 STI screening and treatment at first ANC visit

In the “status quo” scenario, curable STIs (specifically CT, NG and TV) are treated using syndromic management at first ANC clinic attendance. Pregnant women are diagnosed and treated for STIs only if they present with symptoms at the ANC visit. In the alternative “POC screening” scenario, we assume POC diagnostic testing for all pregnant women presenting for first ANC visit, regardless of whether the woman presents with STI symptoms or not. The hypothetical POC is assumed to have 100% sensitivity, and we assume that 92% of diagnosed STIs are effectively treated during pregnancy with an uncertainty range of 85−100%, based on evidence from a previous study that utilized POC testing for curable STIs in antenatal setting [45]. We set assumed STI prevalences based on a 2019 study of pregnant women in Cape Town [9], which found similar STI prevalence to other South African studies in ANC clinics [10, 11]. In this study, the Gene‐Xpert CT/NG assay and the Gene‐Xpert TV assay were used to screen for CT, NG and TV at first ANC visit. The sensitivity and specificity of Gene‐Xpert ranges from 98% to 100% [8], which is comparable to the assumed hypothetical POC of 100%. Although no test achieves true 100% sensitivity in real‐world settings, we aim to simulate an ideal scenario to assess the maximum potential benefits of accurate diagnosis and treatment of curable STIs during pregnancy.

### 2.2 Inputs from the Thembisa model

We adopted several assumptions and outputs from the Thembisa model (version 4.5) for 2022 [46]. Thembisa is a compartmental model of the South African population and HIV epidemic, designed to answer policy questions relating to HIV prevention and treatment. The model is age‐ and sex‐structured, and the HIV epidemic is simulated dynamically from 1985. The model has previously been used to simulate the impact of different HIV programmes in South Africa on vertical transmission of HIV [47].

### 2.3 Effect of STIs on vertical transmission of HIV

We conducted a review and meta‐analysis of studies that examined the association between curable STIs during pregnancy and vertical transmission of HIV. A more detailed description of the methods and results is provided in the Supplementary Materials. The meta‐analysis included 20 studies with specific details provided in Table S1. The pooled relative risk of vertical HIV transmission is 1.75 (95% confidence interval [CI]: 1.54–1.99) in the presence of any curable STI (Figure S1).

### 2.4 Effect of curable STIs on adverse birth outcomes

We synthesized the results from systematic reviews of the associations between STIs and four adverse pregnancy outcomes: stillbirth, PTD, SGA and LBW. We identified three meta‐analyses on the association of curable STIs and adverse birth outcomes each covering different curable STIs, CT [29], NG [33] and TV [34]. Given the similarities in the pooled odds ratios for different STIs, we pooled the odds ratios from the different meta‐analyses to obtain a mean effect of curable STIs for each of the adverse birth outcomes, as shown in Table 1 and Table S2.

### 2.5 Uncertainty analysis and sensitivity analysis

For each of the key parameters of interest, an uncertainty range was specified, and a one‐way sensitivity analysis was conducted to assess the influence of the model parameter on the outcomes of interest. The uncertainty range was also used to calculate a standard deviation for each parameter, and this was used to parameterize a sampling distribution for each parameter (beta in the case of proportions defined on the interval (0, 1) and gamma in the case of positive‐valued parameters, as shown in Table S6). Uncertainty analysis was conducted by drawing 1000 parameter combinations from these sampling distributions, and for all outcomes, 95% confidence intervals and means were calculated from the results obtained using each parameter combination.

### 2.6 Comparison of model results with trial data

We compared the results from our model, to trial data from the Rakai trial from Uganda [38], the WANTAIM trial from Papua New Guinea [40] and Maduo trial from Botswana [42].

## 3 RESULTS

### 3.1 Vertical transmission of HIV

**Figure 1:**
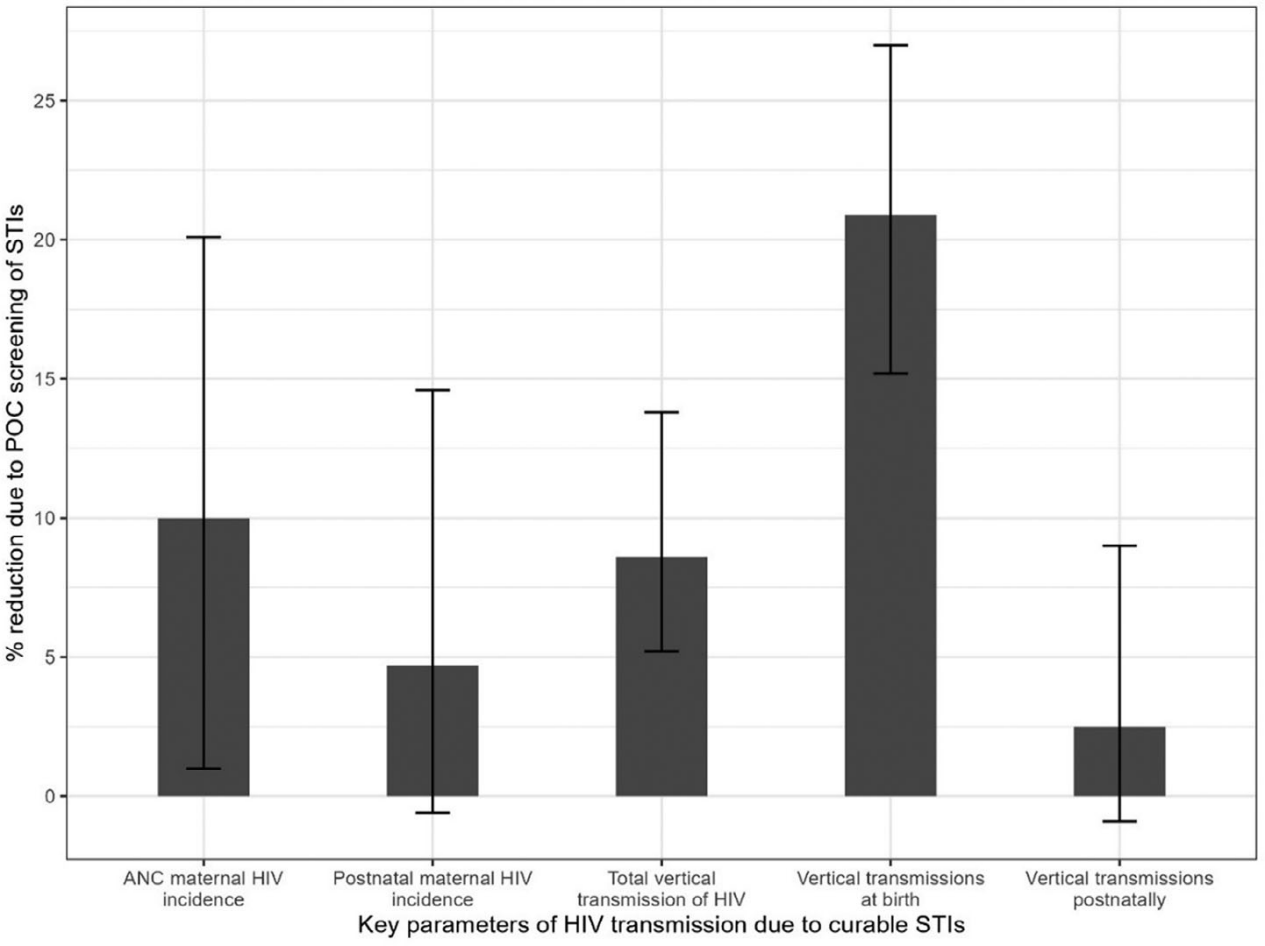
Impact of POC screening of curable STIs in antenatal care on vertical transmission of HIV. Bars represent the percent reduction for each outcome with lower and upper error bars representing confidence intervals. Abbreviations: ANC, antenatal clinic; POC, point‐of‐care; STIs, sexually transmitted infections.

**Figure 2:**
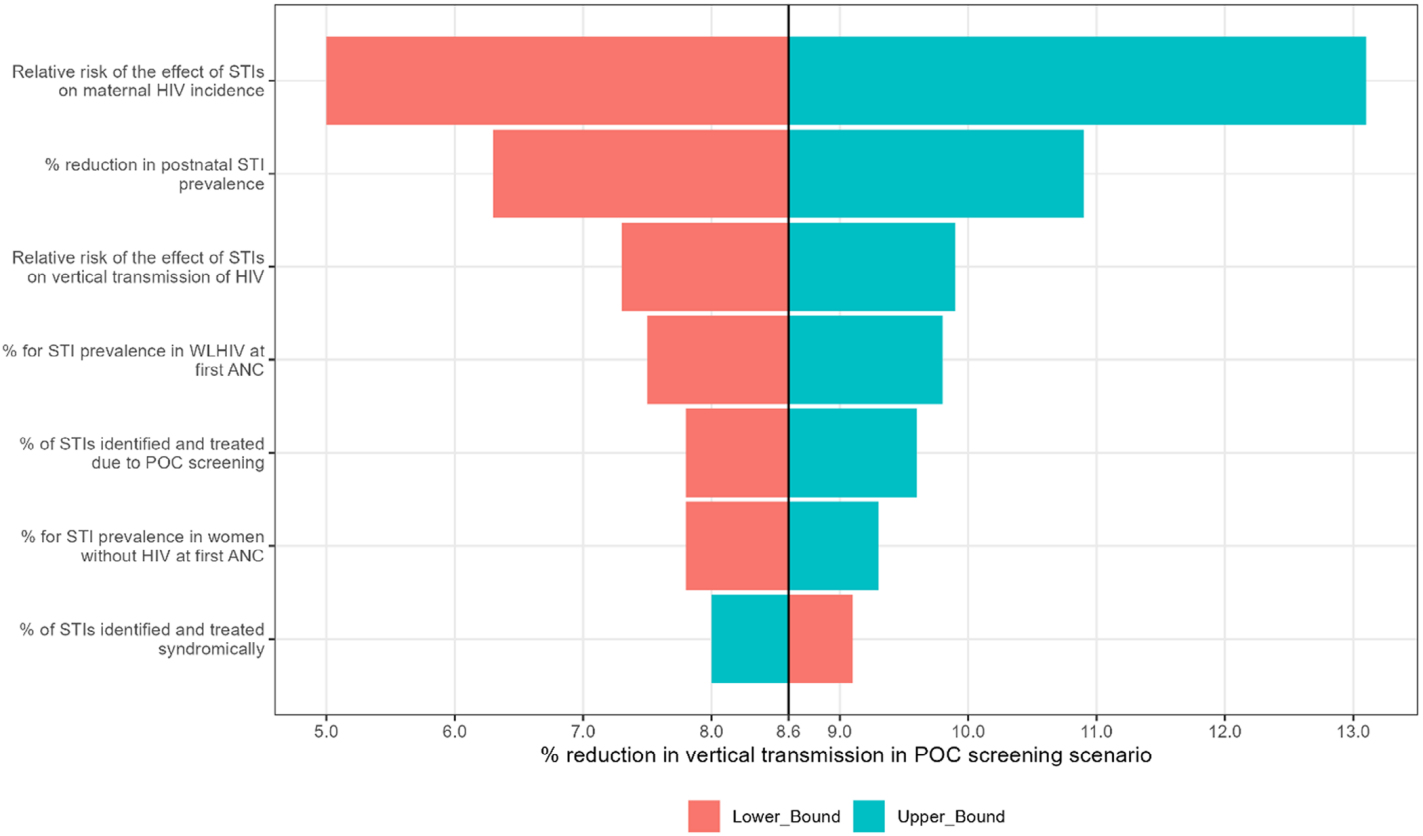
Sensitivity analysis of the impact of POC STI screening in ANC on vertical transmission of HIV. The horizontal bold line represents a reduction in total vertical HIV transmission (8.6%). Abbreviations: ANC, antenatal clinic; POC, point‐of‐care; STIs, sexually transmitted infections; WLHIV, women living with HIV.

The expected reduction in maternal incidence of HIV and vertical transmission of HIV at birth and during breastfeeding in South Africa for the year 2022 is summarized in Table 2 and Figure 1. In the standard of care scenario, using syndromic management for screening of curable STIs (CT, NG and TV), 25.5% of women without HIV and 34.6% of WLHIV will have untreated STIs due to asymptomatic infection at delivery (infections not detected during pregnancy). Overall, untreated curable STI prevalence by the time of delivery is estimated to be 27.8% with syndromic management, as detailed in the Supplementary Material in Section 5. In the POC diagnostic screening scenario, there was a 10.0% (95% CI; 1.0−20.1%) reduction in maternal HIV incidence antenatally and a 4.7% (−0.6% to 14.6%) reduction during the postnatal period. The numbers of vertical transmissions of HIV at birth and postnatally are expected to reduce by 20.9% (15.2–27.0%) and 2.5% (−0.09% to 9.0%), respectively. Overall, the vertical transmission risk of HIV is estimated to reduce by 8.6% (5.2−13.8%) in the POC screening scenario compared to the current syndromic management (Table 2). Figure 2 shows the results of a sensitivity analysis, in which key model parameters were varied between lower and upper bounds, as tabulated in the Supplementary Material (Table S4). As illustrated in Figure 2, most of the uncertainty in total vertical transmission of HIV is attributed to uncertainty in the effect of STIs on maternal incidence of HIV.

**Table 2:** Expected reduction in vertical transmission of HIV in 2022 due to POC STI diagnostic screening in ANC

Calculations	SM scenario	POC STI diagnostic scenario numbers (and 95% CI)^a^	% reduction in STI diagnostic scenario (and 95% CI)^a^
Maternal incidence of HIV in the STI POC scenario (annual rate^b^)			
Antenatal	0.01067	0.00969 (0.00835−0.01065)	10.0 (1.0−20.1)
Postnatal	0.01067	0.01032 (0.00872−0.01065)	4.7 (−0.6 to 14.6)
No. of VT of HIV at birth	2600	2064 (1924−2211)	20.9 (15.2−27.0)
No. of VT of HIV during breastfeeding	5225	5095 (4964−5267)	2.5 (−0.9 to 9.0)
Total vertical transmission of HIV	7825	7157 (6928−7367)	8.6 (5.2−13.8)

Abbreviations: ANC, antenatal clinic; CI, credible interval; POC, point of care; SM, syndromic management; STI, sexually transmitted infection; VT, vertical transmission.

^a^ The mean and 95% credible intervals are presented.

^b^ The unit of measurement for incidence is annual rate of acquiring HIV, per HIV‐negative woman.

### 3.2 Adverse pregnancy and birth outcomes

**Figure 3:**
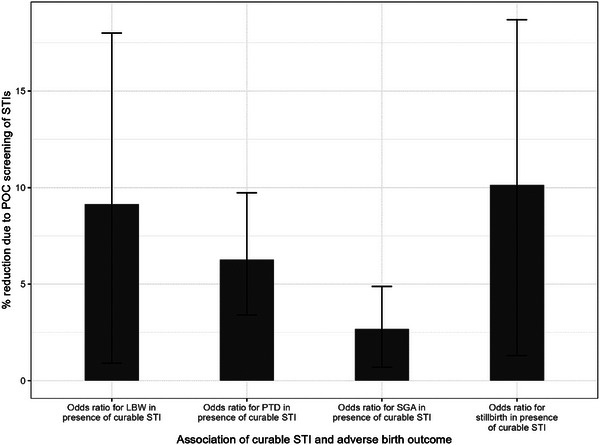
Sensitivity analysis of the impact of POC STI screening in ANC on adverse birth outcomes. Lower and upper error bars are calculated using the 95% confidence interval limits for the odds ratio for the STI‐adverse pregnancy outcome association. Abbreviations: LBW, low birthweight; POC, point‐of‐care; PTD, preterm delivery; SGA, small for gestational age; STIs, sexually transmitted infections.

The probability of adverse birth outcomes differs among women with STIs compared to those without STIs: 2.7% versus 1.7%, respectively, for stillbirth, 14.0% versus 12.1% for PTD, 16.7% versus 14.0% for SGA and 11.8% versus 9.1% for LBW, as calculated in the Supplementary Material in Section 5 (Table S3). POC screening and treatment of curable STIs is estimated to reduce the frequency of stillbirths by 10.14%, PTD by 6.27%, SGA by 2.68% and LBW by 9.19% (Table 3). In Figure 3, we present a sensitivity analysis of the percent reduction in each adverse birth outcome due to POC screening of curable STIs, as tabulated in the Supplementary Material (Table S5). The figure shows wide error bars for LBW and stillbirth due to uncertainty in the effect of curable STIs on the occurrence of LBW and stillbirth in the model.

**Table 3:** Expected reduction in adverse birth outcomes in 2022 due to POC diagnostic screening of STIs in ANC

	Syndromic management (and 95% CI)^a^	POC diagnostic screening (and 95% CI)^a^	% reduction in STI screening scenario (and 95% CI)^a^
**Adverse pregnancy outcome**			
Stillbirths	22,990 (17,475–30,366)	20,658 (15,841–26,132)	10.1 (1.3−18.7)
PTD (exclude stillbirth)	140,811 (132,989–148,627)	131,988 (118,048–143,207)	6.3 (3.4−9.7)
SGA	167,847 (158,182–176,114)	163,345 (154,392–174,059)	2.7 (0.7−4.9)
LBW	120,534 (112,986–127,848)	109,793 (98,734–119,523)	9.1 (0.9−18.0)

Abbreviations: ANC, antenatal clinic; LBW, low birthweight; PTD, preterm delivery; SGA, small for gestational age; STI, sexually transmitted infection.

^a^ The mean and 95% credible intervals are presented.

### 3.3 Comparison of model results with trial data

**Figure 4:**
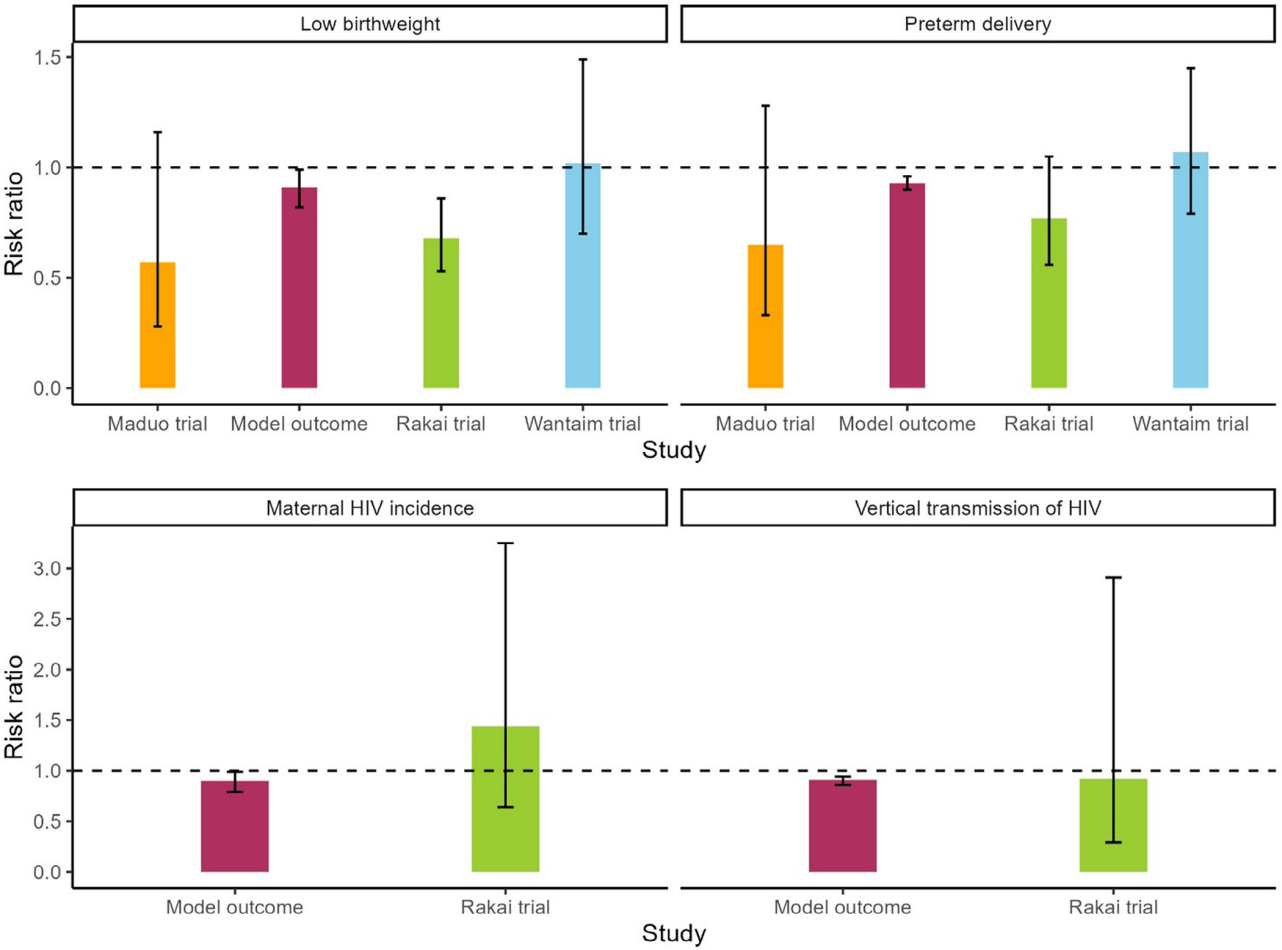
Effect of POC screening and treatment of curable STIs during pregnancy. The dotted lines show the risk ratio of 1. Lower and upper error bars represent the 95% confidence intervals for each outcome. Abbreviations: POC, point‐of‐care; STIs, sexually transmitted infections.

In Figure 4, we present the results from our model, compared to trial data from three different randomized trials. Regarding vertical transmission of HIV, our outcome of a relative risk of 0.91 is comparable to the result in the Rakai trial with a risk ratio of 0.92. For LBW, the risk ratio of 0.91, attributed to POC screening and treatment in our model, falls between the outcomes of the three trials—0.57 from the Maduo trial, 0.68 from the Rakai trial and 1.02 from the WANTAIM trial. Similarly in the case of PTD, our modelled risk ratio of 0.93 falls between the ratios of 0.65 from the Maduo trial, 0.77 from the Rakai trial and 1.07 from the WANTAIM trial.

## 4 DISCUSSION

Our findings suggest that the introduction of POC testing and treatment for the most common curable STIs in South Africa has the potential to reduce vertical HIV transmission and the burden of adverse pregnancy and birth outcomes. This study demonstrates that implementing POC screening and treatment for curable STIs can result in approximately 9% reduction in the vertical transmission of HIV. There is also a reduction in maternal HIV acquisition during the antenatal period (10.0%), as well as a 4.7% reduction in the postnatal period. The introduction of POC screening for curable STIs would also contribute to a decrease in the occurrence of adverse birth outcomes. Estimated reductions were 10.1% for stillbirth, 9.1% for LBW, 6.3% for PTDs and 2.7% for SGA.

The projected decrease of 9% in vertical HIV transmission from our model is moderate but holds promise in advancing efforts towards eliminating vertical HIV transmission. A recent model focusing on pre‐exposure prophylaxis (PrEP) estimated a 5% reduction in vertical transmission with oral PrEP and 24% reduction with injectable PrEP [52]. The provision of comprehensive healthcare services during ANC has the potential to contribute to the elimination of vertical HIV transmission. Similarly, efforts to reduce maternal HIV acquisition will involve various strategies including providing PrEP to all women without HIV who are at high risk of HIV acquisition, regular HIV testing and counselling as well as POC screening and treatment of STIs.

The model estimates of enhanced STI screening and treatment mostly lie within the 95% confidence intervals around the trial estimates, but because the trial estimates are relatively imprecise due to the small sample size, they suggest relatively few statistically significant impacts. Our model suggests that although the intervention has a positive impact on vertical HIV transmission and adverse birth outcomes, the impact is relatively modest, and randomied controlled trials would need to recruit much larger numbers of women if they were to be adequately powered to detect such effects. For the WANTAIM trial, it is also worth noting a significant reduction in curable STIs, particularly CT and TV, in the control group over the course of the trial. This reduction was attributed to the high uptake of sulphadoxine‐pyrimethamine for malaria prophylaxis and could have impacted the overall trial outcomes [40].

There exists a complex interaction between HIV and other curable STIs. Previous studies have shown that the presence of curable STIs can elevate the risk of both acquiring and transmitting HIV [14−16]. In pregnant women, this elevated risk can contribute to an increased incidence of maternal HIV. Our results suggest that the introduction of POC screening for curable STIs in ANC may reduce maternal HIV incidence by 10.0% during pregnancy and 4.7% during breastfeeding. This is estimated to reduce vertical transmission of HIV by 8.6%. A study in South Africa evaluating the effectiveness of the vertical transmission programme of HIV noted that vertical transmission was higher from mothers who had recently acquired HIV during pregnancy or breastfeeding [53]. To achieve the elimination of vertical HIV transmission, there is the need to first reduce maternal HIV incidence, which can be reduced by offering comprehensive maternal healthcare services that include screening and treating for HIV and curable STIs and offering HIV prevention services. HIV and STI coinfection in pregnant women has been shown to increase the risk of vertical transmission of HIV [11, 18]. As a result, it is critical to ensure that all curable infections are diagnosed and promptly treated during pregnancy.

In our model, we assumed no re‐infection with curable STIs during the antenatal period, which is a limitation, as we found persistent STI in 16% of women who had curable STI at first antenatal visit in our previous study [12]. As such, our results represent an ideal scenario in which programmes are able to prevent antenatal re‐infection. However, we did account for the possibility of re‐infection in the postnatal period, assuming an average 50% waning of the protection achieved during the antenatal period. It is important to note that our analysis did not explicitly incorporate partner notification or management interventions, a critical factor in mitigating the risk of re‐infection among women during pregnancy. Previous studies have shown that although over 95% of index cases are willing to notify sexual partners, the proportions of successful partner notification vary from 23% to 95% [54−56]. Partner notification management, though not incorporated into our modelling, has the potential to further alleviate the burden of curable STIs during pregnancy.

The current analysis assumed POC screening test specifications consistent with Gene‐Xpert CT/NG and TV rapid POC. Gene‐Xpert CT/NG and TV tests have a substantially higher cost than syndromic management [57]. Training and operational cost associated with implementing Gene‐Xpert CT/NG and TV tests may be prohibitive for healthcare facilities, especially in resource‐constrained environments where access to laboratory facilities is also limited. In contrast, syndromic management algorithms are simple, inexpensive and easy to apply. We acknowledge that there is still a need to assess the cost‐effectiveness of POC screening relative to syndromic management for CT, NG and TV. Developing cheaper and affordable POC diagnostic tests that are easily accessible and can seamlessly be integrated into routine ANC is urgently needed. This will improve access to screening and treatment for curable STIs among pregnant women.

This analysis has several strengths. First, our analysis integrated data from different sources and synthesized the evidence to show the benefits of diagnostic screening and treatment of curable STIs in ANC. An additional significant contribution of this analysis is our comprehensive review and meta‐analysis of the effect of STIs on vertical transmission of HIV, which has not previously been systematically reviewed. Our analysis also has limitations. First, our assumptions regarding the impact of STIs on vertical transmission of HIV and adverse pregnancy outcomes rely on observational data and there are many possible confounding factors that could explain the observed associations. Second, we used a simple static model which does not consider how reductions of STIs in pregnancy could impact the prevalence of STIs at the population level. A prior modelling study demonstrated that introducing POC screening and treatment of curable STIs among pregnant South African women could lead to a reduction in STI prevalence in the broader population over 10 years (about 7% for CT and 0.3% for NG) [58]. Third, in our analysis, there is uncertainty regarding the extent to which antenatal reductions in STI prevalence are sustained in the postnatal breastfeeding period given the rate of STI re‐infection and incidence in this population. Lastly, the generalizability of our findings to settings beyond South Africa may be limited, primarily due to differences in fertility rates, prevalence of HIV and STIs, and the availability of ANC services.

## 5 CONCLUSIONS

Curable STIs are common among pregnant women in South Africa. POC STI screening and treatment may modestly reduce maternal HIV incidence, vertical HIV transmission, and the risk of adverse pregnancy and birth outcomes and would substantially reduce the burden of curable STIs in pregnancy. The study provides evidence to move beyond the syndromic management of STIs in South Africa, particularly in ANC.

## COMPETING INTERESTS

The authors declare no competing interests.

## AUTHORS’ CONTRIBUTIONS

DCN conceptualized this study with guidance from LFJ, DLJ‐D and LM. LFJ designed the spreadsheet mathematical model and performed the analyses, and DCN assisted in setting the model parameters and uncertainty ranges. DCN drafted the manuscript. LFJ, DJD SD‐M and LM provided iterative critical review on the analysis, revised the manuscript and appraised several drafts before approving the final version submitted for publication.

## Supporting information



Supporting information file 1: Supplementary material

## Data Availability

Data sharing is not applicable to this article as no datasets were generated or analysed during the current study.
