# Supplementary material for: The effect of STI screening during pregnancy on vertical transmission of HIV and adverse pregnancy outcomes in South Africa: a modelling study
Source: J Int AIDS Soc. 2025 Jan 26;28(2):e26410. doi: 10.1002/jia2.26410 (PMC11769709; doi:10.1002/jia2.26410)
Supplement: Supplementary file 1 — Supporting information file 1: Supplementary material [file JIA2-28-e26410-s001.docx]

Supplementary material

Table of Contents

[1 Prevalence of STIs during pregnancy 2](#_Toc179372669)

[2 Effect of STIs on vertical transmission of HIV 3](#_Toc179372670)

[2.1 Search strategy 3](#_Toc179372671)

[2.1.1 Study selection 3](#_Toc179372672)

[2.2 Studies included in the meta-analysis. 4](#_Toc179372673)

[2.3 Meta analysis 6](#_Toc179372674)

[3 Modelling the effect of POC screening on vertical transmission of HIV 7](#_Toc179372675)

[4 Modelling effect of POC screening of curable STIs on adverse birth outcomes 13](#_Toc179372676)

[4.1 Association between curable STIs and adverse pregnancy outcomes 13](#_Toc179372677)

[4.2 Modelling effect of STIs on adverse birth outcomes 14](#_Toc179372678)

[5 Additional results 16](#_Toc179372679)

[5.1 Sensitivity analysis for vertical transmission of HIV 16](#_Toc179372680)

[6 References 18](#_Toc179372681)

# Prevalence of STIs during pregnancy

The assumed STI prevalence in pregnant women was based on a study done between January 2018 to January 2019 [3]. In this study, women self-collected vulvovaginal swab specimens using Xpert CT/NG Vaginal/Endocervical specimen collection kits and trained study staff tested the swabs on site for CT, NG, and TV using the Xpert CT/NG assay and the Xpert TV assay [3]. For the model, we used prevalence of curable STIs in pregnancy as found in this study. The antenatal prevalence of HIV at the study setting is around 30% which is consistent with the national antenatal HIV prevalence [4, 5]. The prevalences of curable STIs was also similar to other studies done in South African antenatal clinics in Tshwane District, Gauteng [6] and in KwaZulu Natal [7].

# Effect of STIs on vertical transmission of HIV

We conducted a review and meta-analysis of studies that examined the association of STIs during pregnancy and vertical transmission of HIV. There are no studies that examined the impact of postnatal STIs on vertical transmission of HIV, nor is there any biological plausibility that postnatal STIs increase vertical transmission of HIV, therefore we assumed no effect of maternal STIs on postnatal transmission risk from mothers living with HIV. Forest plots and the overall random-effects pooled estimates were generated using Stata version 14 [8].

## Search strategy

We searched electronic databases for published literature up to June 30, 2022. Medical Subject Headings (MeSH) terms were searched in MEDLINE (via PubMed), EMBASE, EBSCOhost, Google Scholar Africa Wide, Cochrane Central Register of Controlled trials (CENTRAL), Cumulative Index to Nursing and Allied Health Literature (CINAHL), World Health Organization Library Information System (WHOLIS), Web of Science, PDQ (Pretty Darn Quick)- Evidence and Scopus for publications. No restriction was placed on publication language or time frame.

We used the following MeSH terms: -

“sexually transmitted infections” OR “sexually transmitted diseases” OR “STIs” OR “genital tract infections” OR “reproductive tract infections” OR syphilis OR “treponema pallidum” OR gonorrhea OR “Neisseria gonorrhoeae” OR gonorrhoea OR gonococcus OR chlamydia OR “chlamydia trachomatis” OR trichomonas OR “trichomonas vaginalis” OR trichomoniasis OR “mycoplasma genitalium” OR “m genitalium” OR “m. genitalium” OR “bacterial vaginosis” OR “reproductive tract infection” **AND**

"AIDS serodiagnosis" OR "HIV Infections" OR HIV OR “HIV seropositivity” OR "human immunodeficiency virus" OR "acquired immunodeficiency syndrome" OR "hiv/aids" **AND**

“mother-to-child” OR “vertical transmission” OR MTCT OR breastfeeding OR “breast feeding”

### Study selection

Primary studies were included if: -

i. The participants were pregnant or breastfeeding women living with HIV (all ages)

ii. Tested for a STI during pregnancy.

iii. Reported use of a diagnostic test for STI (excluding syndromic management)

iv. Reported incidence of vertical transmission of HIV during pregnancy or at birth or during postnatal period.

The following variables were collected for data extraction.

1. prevalence of a STI in pregnant or breastfeeding women
2. type of diagnostic test used for the STI
3. duration of follow up
4. timing of vertical transmission of HIV
5. Unadjusted and adjusted OR, RR for the effect of STIs on vertical transmission of HIV and 95% confidence intervals.
6. Incidence rate of vertical transmission of HIV

## Studies included in the meta-analysis.

The electronic search identified 613 title articles. After removing duplicates, 596 articles remained for screening. Abstract screening was done to 596 studies and 568 were found to be irrelevant to this review and were excluded. Of 28 study abstracts identified, 20 were included in the meta-analysis. Eight studies were excluded and the reasons for exclusion were not reporting association of STIs in pregnancy and vertical transmission of HIV, same data points included in another study, no diagnostic testing for STIs and being a narrative review.

| Table S1: Characteristics of included studies on association between STIs during pregnancy and vertical transmission of HIV | | | | | | | |
| --- | --- | --- | --- | --- | --- | --- | --- |
| **Study ID** | **Participants** | **Country** | **Year(s) of study** | **Duration of follow up** | **Type of study** | **STI tested** | **Citation** |
| Adachi 2015 | Pregnant WLHIV | Brazil, SA, Argentina and USA | Apr 2004 - Jul 2010 | Antenatal to 6 months postnatal | Cohort | CT and NG | [9] |
| Aebi-Popp 2016 | Pregnant WLHIV | Ukraine | 2007 - 2012 | Antenatal to 18 months postnatal | Cohort | HSV-2 | [10] |
| Bollen 2008 | Pregnant WLHIV | Thailand | 1996 - 1997 | Antenatal to 3 months postnatal | Cohort | HSV-2 | [11] |
| Calegari 2022 | Pregnant WLHIV | Brazil | Feb 2013 - Feb 2019 | Antenatal to 4 months postnatal | Cross-sectional | Syphilis | [12] |
| Chaisilwattana 1997 | All pregnant women* | Thailand | Jun 1993 - Mar 1994 | Antenatal to 15 months postnatal | Cohort | CT, NG and Syphilis | [13] |
| Chen 2005 | Pregnant WLHIV | USA | Apr 1994 - Apr 1999 | Antenatal to 6 weeks postnatal | Cohort | CT, NG Syphilis and HSV-2 | [14] |
| Cowan 2008 | Pregnant WLHIV | Zimbabwe | 1997 - 2000 | Antenatal to 18 months postnatal | Case-control | HSV-2 | [15] |
| Drake 2007 | Pregnant WLHIV | Kenya | Jul 1999 - Oct 2002 | Antenatal to 12 months postnatal | Cohort | HSV-2 | [16] |
| Farquhar 2010 | Pregnant WLHIV | Kenya | 1999 - 2002 | Antenatal to 12 months postnatal | Cohort | CT, TV, and NG | [17] |
| Fawzi 2000 | Pregnant WLHIV | Tanzania | Apr 1995 - Jul 1997 | Antenatal to 12 months postnatal | Cohort | TV, NG and Syphilis | [18] |
| Gumbo 2010 | Pregnant WLHIV | Zimbabwe | Apr 2002 - Nov 2003 | Antenatal to 15 months postnatal | Cohort | Syphilis and HSV-2 | [19] |
| John 2001 | Pregnant WLHIV | Kenya | Nov 1992 - Oct 1997 | Antenatal to 24 months postnatal | Cohort | CT, TV, NG and Syphilis | [20] |
| Menegotto 2021 | Pregnant WLHIV | Brazil | Feb 2013 - Dec 2016 | Antenatal to 18 months postnatal | Cohort | Syphilis | [21] |
| Mwanyumba 2002 | All pregnant women* | Kenya | Apr 1998 - Apr 1999 | Antenatal to 6 weeks postnatal | Cohort | Syphilis | [22] |
| Mwapasa 2006 | All pregnant women* | Malawi | Dec 2000 - Jun 2004 | Antenatal to 12 weeks postnatal | Cohort | Syphilis | [23] |
| Roxby 2019 | Pregnant WLHIV | Kenya | 1999 - 2005 | Antenatal to 4 weeks postnatal | Case-control | CT, TV, NG, MG, Syphilis | [24] |
| Temmerman 1995 | Pregnant WLHIV | Kenya | Jan 1991 - Mar 1992 | Antenatal to 3 months postnatal | Cohort | CT, NG and Syphilis | [25] |
| Thorne 2008 | Pregnant WLHIV | Ukraine | 2003 - 2005 | Antenatal to 3 months postnatal | Cohort | Syphilis | [26] |
| Van Dyke 1998 | Pregnant WLHIV | United States | Apr 1993 - Mar 1995 | Antenatal to 12 months postnatal | Cohort | CT, Syphilis and HSV-2 | [27] |
| Yeganeh 2015 | Pregnant WLHIV | Brazil, SA, Argentina and USA | Apr 2004 - Jul 2010 | Antenatal to 6 months postnatal | Cohort | Syphilis | [28] |
| *Vertical HIV transmission calculated in WLHIV | | | | | | | |

## Meta analysis

Three time points, intrauterine, intrapartum, and perinatal were included in this meta-analysis (Figure S1). Sub-group analysis was carried out to see if the risk of vertical HIV transmission varied by timing of exposure.

*Intrauterine period -* the pooled relative risk for vertical transmission of HIV during the intrauterine period was 2.70 with 95% confidence interval: 95% CI (1.99 – 3.66).

*Intrapartum period -* the pooled relative risk for vertical transmission of HIV during the intrapartum period was 1.65: 95% CI (1.99 – 3.66).

*Perinatal period -* the pooled relative risk for vertical transmission of HIV during the perinatal period was 1.58: 95% CI (1.36 – 1.85).

The overall pooled relative risk was 1.75 with 95% confidence interval (1.54 – 1.99) and this is the parameter we have used in the model. Average rates for vertical transmission of HIV were adjusted with this relative risk of the effect of curable STIs during pregnancy on vertical transmission of HIV as defined in the model equations in the following Section 4.


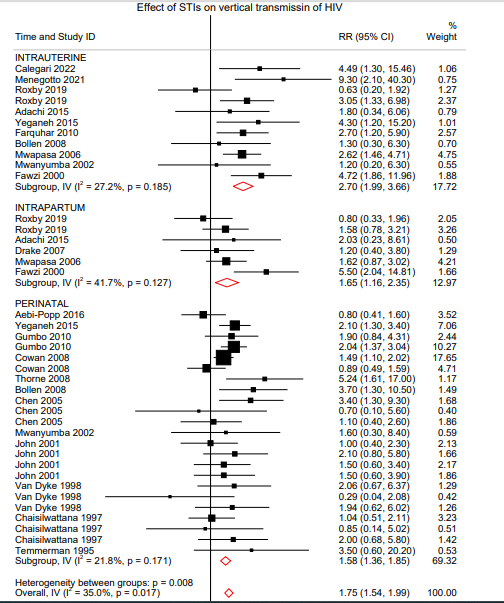


Figure S1: Forest plots for the effect of curable STIs on vertical transmission of HIV

# Modelling the effect of POC screening on vertical transmission of HIV

The model allows for two types of vertical transmission of HIV: perinatal transmission (at or before the time of birth, i.e. intrapartum or intrauterine) and postnatal transmission (transmission occurring due to breastfeeding). We define the following symbols using estimates from the Thembisa 4.5 estimates for 2022 [1]:

J_0_(t) = total number of births, in year t, to all women;

J_1_(t) = number of births, in year t, to WLHIV;

J_2_(t) = number of WLHIV who were on ART at conception;

Se = sensitivity of HIV screening algorithm used in pregnant women (excluding women in the window period from the denominator);

T_1_ = average gestation (in weeks) at which women first seek antenatal care;

T_2_ = average gestation (in weeks) at which women are offered rescreening;

T_3_ = average gestation (in weeks) at which women deliver;

The value of Se has been set at 0.975, reflecting the variable performance of rapid HIV testing algorithms in South Africa [29-32]. The values of T_1_ and T_3_ have been set at 23 weeks and 39 weeks respectively, and the assumed average duration at rescreening T_2_ is 34 weeks.

ν_0_ = Annual HIV incidence rate in pregnant and breastfeeding women, in status quo scenario (using syndromic management for curable STIs)

ν_1_ = Increase in vertical transmission of HIV risk at birth if mother has untreated STI (relative risk)

ν_2_ = Increase in maternal HIV incidence rate if mother has untreated STI (relative risk)

π_0_ = proportion of untreated women tested for HIV at first antenatal visit set at 0.98

π _1_ = probability of being tested later in pregnancy if missing first ANC arbitrarily set to 0.475

π _2_ = probability of being retested later in pregnancy if tested negative at first ANC set to 0.76

π _3_ = proportion of newly diagnosed mothers who start ART during pregnancy set as 0.95

s_1_ = proportion of pregnant women without HIV who are positive for chlamydia, gonorrhoea or trichomoniasis at first ANC

s_2_ = proportion of pregnant WLHIV who are positive for chlamydia, gonorrhoea or trichomoniasis at first ANC

s_3_ = proportion of pregnant women with curable STIs who are identified and treated for chlamydia, gonorrhoea or trichomoniasis at first ANC syndromically

s_4_ = percentage of reduction in STI prevalence in the breastfeeding period due to aetiological screening arbitrary assumed to be 50%

s_5_ = proportion of pregnant women with curable STIs who are identified and treated for chlamydia, gonorrhoea or trichomoniasis at first ANC with POC screening

p_1_ = average rate of vertical transmission of HIV at birth in WLHIV not on ART

p_2_ = average rate of vertical transmission of HIV at birth in acutely infected WLHIV

p_3_ = average rate of vertical transmission of HIV in WLHIV on ART before conception

p_4_ = average rate of vertical transmission of HIV in WLHIV starting ART during pregnancy

To calculate the number of births in year t to:

1. HIV negative women at first ANC visit defined as J_3_(t):

J_3_(t) =(J_0_(t) - J_1_(t)) / (1- ν_0_*(T_3_ – T_1_)/52)

1. Untreated WLHIV at 1^st^ ANC visit defined as J_4_(t)

J_4_(t) = J_0_(t) – J_3_(t) – J_2_(t)

1. Untreated WLHIV in window period who seroconverted during pregnancy J_5_(t).

J_5_(t) = J_3_(t)*(ν_0_*4/52)/ (1 - ν_0_*4/52)

Assuming that only symptomatic infections will be identified in the syndromic scenario (with s_3_ offset to 9% of all women with a curable STI [3]), we calculate the prevalence of infections that remain untreated during pregnancy as J_6_(t) in women without HIV and J_7_(t) in WLHIV defined as:

1. J_6_(t) = s_1_*(1- s_3_)
2. J_7_(t) = s_2_*(1- s_3_)

Assuming that some of the women testing positive for curable STI in the POC screening do not receive treatment during pregnancy, we set the proportion of women identified and treated to s_4_ offset to 92% [3, 6, 7], we calculate the prevalence of infections that remain untreated during pregnancy in the POC screening scenario as J_8_(t) in women without HIV and J_9_(t) in WLHIV defined as:

1. J_8_(t) = s_1_*(1- s_4_)
2. J_9_(t) = s_2_*(1- s_4_)

The 4 in the equation above is the assumed window period on standard antibody tests [33]. The period of 4 weeks is added to reflect the fact that some women who are HIV seronegative at their first antenatal visit will in fact be in the window period.

Maternal incidence rate during antenatal in the POC screening of STIs scenario I(t) in year t is defined as:

I(t) = ν_0_/ (1 + J_6_(t) *(ν_2_ – 1)) *(1 + J_6_(t)) *(1-s_5_) *(ν_2_ – 1)

Maternal incidence rate during postnatal in the POC screening of STIs scenario H(t) in year t is defined as:

H(t) = (I(t)*(1 + J_6_(t) *(1 – s_4_)) *(ν_2_ – 1)))

To calculate vertical transmission of HIV in the POC screening of STIs scenario the following variables are calculated:

Average vertical transmission of HIV at birth in untreated WLHIV q_1_:

1. q_1_= p_1_ / (1 + J_9_*(ν_2_ – 1)) *(1 + J_9_ *(1 – s_5_) *(ν_2_ – 1))

Average vertical transmission of HIV at birth in acutely infected WLHIV q_2_:

1. q_2_= p_2_/ (1 + J_9_*(ν_2_ – 1)) *(1 + J_9_ *(1 – s_5_) *(ν_2_ – 1))

Average vertical transmission of HIV at birth in WLHIV on ART before conception q_3_:

1. q_3_= p_4_/ (1 + J_9_*(ν_2_ – 1)) *(1 + J_9_ *(1 – s_5_) *(ν_2_ – 1))

Average vertical transmission of HIV at birth in WLHIV who start ART antenatally q_4_:

1. q_4_= p_3_/ (1 + J_9_*(ν_2_ – 1)) *(1 + J_9_ *(1 – s_5_) *(ν_2_ – 1))

Probability that untreated WLHIV at first ANC starts ART during pregnancy is calculated as π_4_:

1. π_4_= (π_0_ *Se + (1 - π_0_) * π_1_*Se + π_0_*(1 – Se) * π_2_*Se) * π_3_

Probability of seroconversion between first ANC visit and retesting visit π_5_ is calculated as follows:

1. π_5_ = ν_0_ *(T_2_ – T_1_)/52

Probability of seroconversion between first ANC visit and retesting visit in the POC screening of STIs scenario π_6_ is calculated as follows:

1. π_6_ = I(t)*(T_2_ – T_1_)/52

Probability of diagnosis for women who seroconvert between first ANC visit and 34 weeks π_7_ is calculated as follows:

1. π_7_= (π_0_ * π_2_ +(1 – π_0_) * π_1_) *Se

Probability of seroconversion between retesting and 4 weeks postpartum π_8_ is calculated as follows:

1. π_8_ = ν_0_ *(T_3_ + 4 – T_2_)/52

Probability of seroconversion between retesting and 4 weeks postpartum in the POC screening of STIs scenario π_9_ is calculated as follows:

1. π_9_= I(t) *(T_3_ + 4 – T_2_)/52

Probability of postnatal vertical transmission in WLHIV not on ART π_10_ is set as 18.6%, calibrated to match Thembisa estimates of vertical transmission.

Probability of postnatal vertical transmission in acutely infected WLHIV not on ART is set as π_11_= 27% [34].

Probability of postnatal vertical transmission in WLHIV who started ART antenatally π_12_ is set as 0.0102 based on a monthly Thembisa estimate (0.0017) and assuming 6 months duration of breast feeding.

Probability of postnatal vertical transmission in WLHIV on ART prior conception π_13_ is set as 0.0018 based on a monthly Thembisa estimate (0.0003) and assuming 6 months duration of breast feeding.

Average duration of breastfeeding in women without HIV π_14_ is set as 15.6 months.

Total number of WLHIV who will be on ART at delivery sums up annual number of WLHIV on ART at conception and number of WLHIV who start ART during pregnancy.

Number of WLHIV at first ANC visit who start ART during pregnancy given as ω_1_:

1. ω_1_ = π_4_* (J_3_(t) – J_4_(t))

Number of women without HIV at first ANC visit who seroconverted during pregnancy and start ART during pregnancy given as ω_2_:

1. ω_2_ = (J_5_(t) + J_3_(t)* π_5_) * π_7_* π _3_

Number of women without HIV at first ANC visit but seroconverted during pregnancy and start ART at during pregnancy in the POC screening of STIs scenario is given as ω_3_:

1. ω_3_ = (J_5_(t) + J_3_(t)* π_6_) * π_7_* π _3_

Number of WLHIV who were seropositive at first ANC and do not start ART during pregnancy ω_4_:

1. ω_4_ = (1 - π_4_) *(J_3_(t)

Number of WLHIV who were seronegative at first ANC, seroconverted during pregnancy and do not start ART during pregnancy ω_5_:

1. ω_5_ = (J_4_(t) + J_2_(t)* π_5_) * (1 – π_7_* π _3_) + J_2_(t)* π_8_

Number of WLHIV who were seronegative at first ANC, seroconverted during pregnancy and do not start ART during pregnancy and in the POC screening of STIs scenario ω_6_:

1. ω_6_ = (J_4_(t) + J_2_(t)* π_6_) * (1 – π_7_* π _3_) + J_2_(t)* π_9_

Number of vertical transmissions at birth to women with different HIV and ART status given as:

1. Transmissions from WLHIV and on ART before conception f_1_ given as:

f_1_ = J_2_(t)*p_3_

1. Transmissions from WLHIV and on ART before conception in the POC screening of STIs scenario f_2_ given as:

f_2_ = J_2_(t)*q_3_

1. Transmissions from WLHIV who start ART during pregnancy f_3_ given as:

f_3_ = (ω_1_ + ω_2_) * p_4_

1. Transmissions from WLHIV who start ART during pregnancy in the POC screening of STIs scenario f_4_ given as:

f_4_= (ω_1_ + ω_3_) * q_4_

1. Transmissions from WLHIV who do not start ART during pregnancy f_5_ given as:

f_5_= ω_4_* p_1_ + ω_5_* p_2_

1. Transmissions from WLHIV who do not start ART during pregnancy in the POC screening of STIs scenario f_6_ given as:

f_6_= ω_4_* q_1_ + ω_6_* q_2_

Total number of vertical transmissions of HIV at birth Y_0_(t)= f_1_ + f_3_ + f_5_

Total number of vertical transmissions of HIV at birth in the POC screening of STIs scenario Y_2_(t)= f_2_ + f_4_ + f_6_

Number of postnatal vertical transmissions of HIV from women with different HIV and ART status given as:

1. Transmission in WLHIV on ART before conception r_1_ given as:

r_1_ = (J_3_(t) – f_1_) * π_13_

1. Transmission in WLHIV on ART before conception in the POC screening of STIs scenario r_2_:

r_2_ = (J_3_(t) – f_2_) * π_13_

1. Transmission in WLHIV who start ART during pregnancy r_3_ given as:

r_3_ = (ω_1_ + ω_2_ - f_3_) * π_12_

1. Transmission in WLHIV who start ART during pregnancy in the POC screening of STIs scenario r_4_ given as:

r_4_ = (ω_1_ + ω_3_ – f_4_) * π_12_

1. Transmission in WLHIV who do not start ART during pregnancy r_5_ given as:

r_5_= (ω_4_ + ω_5_ - f_5_) * π_10_

1. Transmission in WLHIV who do not start ART during pregnancy in the POC screening of STIs scenario r_6_ given as:

r_6_= (ω_4_+ ω_6_ – f_6_) * π_10_

1. Transmission in WLHI who seroconvert postnatally r_7_ given as:

r_7_= (J_0_(t) - J_1_(t)) *(1 – exp (-ν_0_* π_14_/12))* π_11_

1. Transmission in WLHI who seroconvert postnatally in the POC screening of STIs scenario r_8_:

r_8_= J_2_(t)*(1 - I(t)*(T_3_ – T_1_)/52) *(1 – exp (-H(t)* π_14_/12)) * π_11_

Total number of postnatal vertical transmissions in the status quo scenario Y_3_(t)is calculated as follows:

Y_3_(t) = r_1_ + r_3_ + r_5_ + r_7_

Total number of postnatal vertical transmissions in the POC screening of STIs scenario Y_4_(t) is calculated as follows:

Y_4_(t) = r_2_+ r_4_ + r_6_ + r_8_

Total number of vertical transmissions of HIV in the status quo scenario is sum of Y_0_(t) and Y_3_(t)

Total number of vertical transmissions of HIV in the POC screening of STIs scenario is sum of Y_2_(t) and Y_4_(t)

# Modelling effect of POC screening of curable STIs on adverse birth outcomes

## Association between curable STIs and adverse pregnancy outcomes

We assessed the effect of STIs on the prevalence of four adverse pregnancy outcomes: stillbirth, pre-term delivery (PTD), small for gestational age (SGA) and low birthweight (LBW). We identified three meta-analyses on the association of curable STIs and adverse birth outcomes [35-37]. Olson-Chen and colleagues presented a meta-analysis in 2018 on the association between *Chlamydia trachomatis* (CT) and adverse birth outcomes which included PTD, SGA and LBW [35]. In 2020, Valley and colleagues performed a systematic review and meta-analysis on the association of adverse pregnancy and neonatal outcomes with *Neisseria gonorrhoeae* (NG) [36], assessing PTD and LBW. In 2021, Van Gerwen and colleagues evaluated the association between *Trichomoniasis vaginalis* (TV) and adverse birth outcomes, including PTD and LBW [37]. There were similarities in the odds ratios presented from the three meta-analyses assessing the effects of the three different STIs (CT, NG, TV). Therefore, we pooled the odds ratios from the different meta-analyses by performing a meta-analysis to obtain a mean effect of curable STIs, for each of the adverse birth outcomes, as shown in Table S1. The mean effects are included in Table 1 of the main paper.

| Table S2: Effect of STIs on the prevalence of adverse birth outcomes | | | | | |
| --- | --- | --- | --- | --- | --- |
| Adverse event | Odd ratio source | STI assessed | Odds ratio | 95% LL | 95% UL |
| Stillbirth | Olson-Chen et al (2018) | CT | 1.44 | 1.06 | 1.94 |
|  | Vallely et al (2021) | NG | 2.12 | 0.50 | 8.94 |
|  | **Pooled from meta-analysis** | **CT, NG** | **1.46** | **1.08** | **1.96** |
| Pre-term deliveries (PTD) | Olson-Chen et al (2018) | CT | 1.27 | 1.05 | 1.54 |
|  | Van Gerwen et al (2021) | TV | 1.27 | 1.08 | 1.50 |
|  | Vallely et al (2021) | NG | 1.55 | 1.21 | 1.99 |
|  | **Pooled from meta-analysis** | **CT, TV, NG** | **1.32** | **1.18** | **1.47** |
| Small for gestational age (SGA) | Olson-Chen et al (2018) | CT | 1.14 | 1.05 | 1.25 |
| Low birthweight (LBW) | Olson-Chen et al (2018) | CT | 1.34 | 1.21 | 1.48 |
|  | Van Gerwen et al (2021) | TV | 2.12 | 1.15 | 3.91 |
|  | Vallely et al (2021) | NG | 1.66 | 1.12 | 2.48 |
|  | **Pooled from meta-analysis** | **CT, TV, NG** | **1.47** | **1.06** | **2.05** |

## Modelling effect of STIs on adverse birth outcomes

We define the following symbols as stated in the model parameter Table 1 in the main paper:

β_1_ = proportion of pregnancies that result in stillbirth;

β _2_ = proportion of live births that are pre-term delivery (PTD);

β _3_ = proportion of live births that are small for gestation age (SGA);

β _4_ = proportion of live births that are low birth weight (LBW);

α_1_ = odds ratio for stillbirth in the presence of curable STI;

α _2_ = odds ratio for PTD in the presence of curable STI;

α _3_ = odds ratio for SGA in the presence of curable STI;

α _4_ = odds ratio for LBW in the presence of curable STI;

The following parameters are defined and calculated as follows:

1. Total pregnancies which end in live birth or stillbirth J_6_(t) defined as;

J_6_(t) = J_0_(t)/ (1 – β_1_),

1. Prevalence of curable STI at first ANC visit S_x_ is calculated as;

S_x_ = (J_2_(t)*s_1_ + (J_6_(t) - J_2_(t)) *s_2_)/ J_6_(t),

1. Untreated curable STI prevalence by the time of delivery S_d_ is calculated as;

S_d_ = S_x_ *(1 – s_3_)

We used parameters that were defined in section 3 as follows:

- J_0_(t) = the total live births
- J_2_(t) = women who are HIV negative at first ANC visit
- s_1_ = proportion of pregnant women without HIV who are positive for chlamydia, gonorrhoea or trichomoniasis at first ANC
- s_2_ = proportion of pregnant WLHIV who are positive for chlamydia, gonorrhoea or trichomoniasis at first ANC
- s_3_ = proportion of pregnant women with curable STIs who are identified and treated under current syndromic management
- φ_1_ = proportion of pregnancies which end in stillbirth in the population

There is a direct relationship between relative risk and odds ratio which is linked by the proportion of pregnancies that result in an adverse birth outcome if there are no STIs present which is the Base Rate, x_1_.

The Odds Ratio can be expressed in terms of Relative risk and the base rate as follows:

| Odds Ratio | = | Relative risk x | 1 - Base Rate |
| --- | --- | --- | --- |
|  |  |  | 1- Relative risk x Base Bate |

This can be re-arranged as follows:

| Relative risk | = | Odds Ratio |
| --- | --- | --- |
|  |  | 1 – Base Rate x (1 - Odds Ratio) |

Let Base Rate of stillbirth in women with no STIs, x_1_, be determined from the total percentage rate of pregnancies that result in stillbirth (φ_1_), the percentage of pregnant women with STIs in the population and the Odds Ratio.

| Base rate = x_1_ | = | φ _1_ |
| --- | --- | --- |
|  |  | 1 - S_d_ + S_d_ x Relative risk |

The equation can be reduced to:

| Base rate | = | φ _1_ |
| --- | --- | --- |
|  |  | 1 - S_d_ x (1 - Relative risk) |

When we substitute the formula for the relative risk into the above equation, the equation reduces into a quadratic equation, of the form *a*x*_1_*^2^ + *b*x*_1_* + *c* = 0, where *a, b* and *c* are known.

a = (1 - S_d_) x (Odds Ratio -1)

b = 1 + (Odds Ratio - 1) x (S_d_ - φ _1_) = 1 – (Odds Ratio -1) x (φ _1_ - S_d_)

c = Prevalence of stillbirth among South African pregnant women = φ _1_

Solving the quadratic, we calculate Base rate = x_1_ = $\frac{-b+\sqrt{b^{2}-4ac}}{2a}$

We repeated this process for each of the adverse birth outcomes to calculate the Base Rate of the adverse birth outcome in women with no STI (Table S3). The values shown in Table S3 are those obtained when using the mean STI prevalence levels and odds ratios in Table 1 of the main text (results change in the uncertainty analysis when sampling different values of these parameters).

We further calculate the probability of experiencing an adverse outcome in the presence of STI defined as x_d_

For still birth:

| x_d_ | = | 1 |
| --- | --- | --- |
|  |  | [(1 + (1 - x_1_) /(x_1_ * α_1_)] |

We perform the same process for all the adverse birth outcomes to calculate the probability of experiencing adverse birth outcome in the presence of STI, **Table S3.**

| Table S3: Probabilities of occurrence of an adverse birth outcome in pregnant women | | |
| --- | --- | --- |
| **Adverse event** | **Women with no STI** | **Women with STI** |
| Stillbirth | 0.017 | 0.027 |
| PTD | 0.121 | 0.140 |
| SGA | 0.140 | 0.167 |
| LBW | 0.091 | 0.118 |

# Additional results

## Sensitivity analysis for vertical transmission of HIV

**Table S4** shows the results of a sensitivity analysis, in which key model parameters were varied between lower and upper bounds as illustrated in Figure 2 in the main text of the paper.

| Table S4: Sensitivity analysis for vertical transmission of HIV with POC screening and treatment of STIs in pregnancy | | | |
| --- | --- | --- | --- |
|  | Reduction in vertical transmission of HIV - base is 8.60% | | |
| Parameter | Lower bound | Upper bound | Parameter range |
| Increase in HIV vertical transmission risk at birth if mother has untreated STI (RR) | 7.30% | 9.90% | 1.75 (1.54 - 1.99) |
| Increase in maternal HIV incidence rate if mother has untreated STI (RR) | 5.00% | 13.10% | 1.50 (1.20 - 1.80) |
| % of pregnant women without HIV and positive for curable STIs at first ANC | 7.80% | 9.30% | 28% (20% - 36%) |
| % of pregnant WLHIV and positive curable STIs at first ANC | 7.50% | 9.80% | 38% (29% - 49%) |
| % of STIs identified and treated syndromically | 9.10% | 8.00% | 9% (3% - 17%) |
| % of STIs identified and treated in POC scenario | 7.80% | 9.60% | 92% (85% - 100%) |
| % reduction in STI prevalence while breastfeeding, due to aetiological ANC screening | 6.30% | 10.90% | 50% (0% - 100%) |
| Abbreviation: ANC; antenatal clinic, POC; point of care, STI; sexually transmitted infections, WLHIV; women living with HIV | | | |
| Curable STIs refers to CT; Chlamydia trachomatis, NG; Neisseria gonorrhoeae, TV; Trichomonas vaginalis, | | | |

**Table S5** shows the results of a sensitivity analysis, in which we varied the odds ratio for the association between untreated STIS and each adverse birth outcome between lower and upper bounds and assessed the effect on adverse birth outcome. The sensitivity analysis showed wide variation when we adjusted the range the odds ratios between lower and upper bounds for low birthweight 10% with 95% CI (1.2 – 19.6%) and stillbirth 11.1% with 95% CI (2.4 – 20.6%).

| Table S5: Sensitivity analysis for the reduction in adverse birth outcomes with POC screening and treatment of STI in pregnancy | | | | |
| --- | --- | --- | --- | --- |
|  |  | Reduction in adverse birth outcome | | |
|  | Parameter range | Baseline | Lower bound | Upper bound |
| OR for stillbirth if experiencing an CT/NG/TV | 1.46 (1.09 - 1.97) | 11.10% | 2.40% | 20.60% |
| OR for PTD if experiencing an CT/NG/TV | 1.32 (1.18 - 1.48) | 6.90% | 3.90% | 9.90% |
| OR for SGA if experiencing an CT/NG/TV | 1.14 (1.05 - 1.25 | 2.90% | 0.90% | 5.20% |
| OR for LBW if experiencing an CT/NG/TV | 1.48 (1.06 - 2.06) | 10.10% | 1.20% | 19.60% |

Table S6: Key parameters and standard deviations for uncertainty range

| Key parameter | Mean | Standard deviation | Prior type |
| --- | --- | --- | --- |
| Prevalence of STIs in WLHIV | 0.380 | 0.043 | Beta |
| Prevalence of STIs in women without HIV | 0.280 | 0.041 | Beta |
| % of STIs treated syndromically | 0.090 | 0.036 | Beta |
| % of STIs treated under POC | 0.920 | 0.038 | Beta |
| Effect of STIs on vertical transmission of HIV | 1.750 | 0.115 | Gamma |
| Effect of STIs on maternal HIV acquisition | 1.500 | 0.255 | Gamma |
| POC effect on postnatal STI prevalence | 0.500 | 0.289 | Beta |
| Prevalence of stillbirth | 0.020 | 0.002 | Beta |
| Prevalence of PTD | 0.125 | 0.006 | Beta |
| Prevalence of SGA | 0.149 | 0.006 | Beta |
| Prevalence of LBW | 0.107 | 0.004 | Beta |
| Association between STIs and stillbirth | 1.462 | 0.224 | Gamma |
| Association between STIs and PTD | 1.322 | 0.075 | Gamma |
| Association between STIs and SGA | 1.140 | 0.051 | Gamma |
| Association between STIs and LBW | 1.477 | 0.254 | Gamma |
| Abbreviation: ANC; antenatal clinic, POC; point of care, PTD; Pre-term deliveries, LBW; low birthweight, SGA; small for gestational age, STI; sexually transmitted infections, WLHIV; women living with HIV | | | |

# References

1. Johnson LF, Dorrington RE. Modelling the impact of HIV in South Africa’s provinces: 2022 update. Centre for Infectious Disease Epidemiology and Research, University of Cape Town. Available: <https://www.thembisa.org/downloads>. 2022.

2. Peters RP, Garrett N, Chandiwana N, Kularatne R, Brink AJ, Cohen K, et al. Southern African HIV Clinicians Society 2022 guideline for the management of sexually transmitted infections: Moving towards best practice. Southern African journal of HIV medicine. 2022;23(1):1450.

3. Davey DLJ, Nyemba DC, Gomba Y, Bekker L-G, Taleghani S, DiTullio DJ, et al. Prevalence and correlates of sexually transmitted infections in pregnancy in HIV-infected and-uninfected women in Cape Town, South Africa. PloS one. 2019;14(7).

4. National Department of Health. The 2019 National Antenatal Sentinel Survey - Key Findings. In: Health SANDo, editor. 2021.

5. Woldesenbet SA, Kufa T, Barron P, Ayalew K, Cheyip M, Chirombo BC, et al. Assessment of readiness to transition from antenatal HIV surveillance surveys to PMTCT programme data-based HIV surveillance in South Africa: The 2017 Antenatal Sentinel HIV Survey. International Journal of Infectious Diseases. 2020;91:50-6.

6. Mudau M, Peters RP, De Vos L, Olivier DH, J Davey D, Mkwanazi ES, et al. High prevalence of asymptomatic sexually transmitted infections among human immunodeficiency virus-infected pregnant women in a low-income South African community. International journal of STD & AIDS. 2018;29(4):324-33.

7. Moodley D, Moodley P, Sebitloane M, Soowamber D, McNaughton-Reyes HL, Groves AK, et al. High prevalence and incidence of asymptomatic sexually transmitted infections during pregnancy and postdelivery in KwaZulu Natal, South Africa. Sexually transmitted diseases. 2015;42(1):43-7.

8. StataCorp. Stata Statistical Software: Release 15. College Station, TX: StataCorp LLC. 2017.

9. Adachi K, Klausner JD, Bristow CC, Xu J, Ank B, Morgado MG, et al. Chlamydia and Gonorrhea in HIV-Infected Pregnant Women and Infant HIV Transmission. Sex Transm Dis. 2015;42(10):554-65.

10. Aebi-Popp K, Bailey H, Malyuta R, Volokha A, Thorne C. High prevalence of herpes simplex virus (HSV)-type 2 co-infection among HIV-positive women in Ukraine, but no increased HIV mother-to-child transmission risk. BMC pregnancy and childbirth. 2016;16(1):1-9.

11. Bollen LJ, Whitehead SJ, Mock PA, Leelawiwat W, Asavapiriyanont S, Chalermchockchareonkit A, et al. Maternal herpes simplex virus type 2 coinfection increases the risk of perinatal HIV transmission: possibility to further decrease transmission? Aids. 2008;22(10):1169-76.

12. Calegari LH, Friedrich L, Astolfi VR, Kerber JM, Andrades GS, Da Silva CH. The Impact of Maternal Syphilis and Associated Factors on HIV Vertical Transmission. The Pediatric Infectious Disease Journal. 2022;41(7):563-5.

13. Chaisilwattana P, Chuachoowong R, Siriwasin W, Bhadrakom C, Mangclaviraj Y, Young NL, et al. Chlamydial and gonococcal cervicitis in HIV-seropositive and HIV-seronegative pregnant women in Bangkok: prevalence, risk factors, and relation to perinatal HIV transmission. Sex Transm Dis. 1997;24(9):495-502.

14. Chen KT, Segú M, Lumey LH, Kuhn L, Carter RJ, Bulterys M, et al. Genital herpes simplex virus infection and perinatal transmission of human immunodeficiency virus. Obstetrics & Gynecology. 2005;106(6):1341-8.

15. Cowan FM, Humphrey JH, Ntozini R, Mutasa K, Morrow R, Iliff P. Maternal Herpes simplex virus type 2 infection, syphilis and risk of intra-partum transmission of HIV-1: results of a case control study. Aids. 2008;22(2):193-201.

16. Drake AL, John-Stewart GC, Wald A, Mbori-Ngacha DA, Bosire R, Wamalwa DC, et al. Herpes simplex virus type 2 and risk of intrapartum human immunodeficiency virus transmission. Obstetrics & Gynecology. 2007;109(2 Part 1):403-9.

17. Farquhar C, Mbori-Ngacha D, Overbaugh J, Wamalwa D, Harris J, Bosire R, et al. Illness during pregnancy and bacterial vaginosis are associated with in-utero HIV-1 transmission. Aids. 2010;24(1):153-5.

18. Fawzi W, Msamanga G, Renjifo B, Spiegelman D, Urassa E, Hashemi L, et al. Predictors of intrauterine and intrapartum transmission of HIV-1 among Tanzanian women. Aids. 2001;15(9):1157-65.

19. Gumbo FZ, Duri K, Kandawasvika GQ, Kurewa NE, Mapingure MP, Munjoma MW, et al. Risk factors of HIV vertical transmission in a cohort of women under a PMTCT program at three peri-urban clinics in a resource-poor setting. J Perinatol. 2010;30(11):717-23.

20. John GC, Nduati RW, Mbori-Ngacha DA, Richardson BA, Panteleeff D, Mwatha A, et al. Correlates of mother-to-child human immunodeficiency virus type 1 (HIV-1) transmission: association with maternal plasma HIV-1 RNA load, genital HIV-1 DNA shedding, and breast infections. The Journal of infectious diseases. 2001;183(2):206-12.

21. Menegotto M, Magdaleno AM, da Silva CL, Friedrich L, da Silva CH. Mother-to-Child HIV Transmission among Pregnant Women in a City with the Highest Rates of HIV in Brazil. American Journal of Perinatology. 2021;39(13):1418-25.

22. Mwanyumba F, Gaillard P, Inion I, Verhofstede C, Claeys P, Chohan V, et al. Placental inflammation and perinatal transmission of HIV-1. JAIDS Journal of Acquired Immune Deficiency Syndromes. 2002;29(3):262-9.

23. Mwapasa V, Rogerson SJ, Kwiek JJ, Wilson PE, Milner D, Molyneux ME, et al. Maternal syphilis infection is associated with increased risk of mother-to-child transmission of HIV in Malawi. Aids. 2006;20(14):1869-77.

24. Roxby AC, Yuhas K, Farquhar C, Bosire R, Mbori-Ngacha D, Richardson BA, et al. Mycoplasma genitalium infection among HIV-infected pregnant African women and implications for mother-to-child transmission of HIV. Aids. 2019;33(14):2211-7.

25. Temmerman M, Nyong'o AO, Bwayo J, Fransen K, Coppens M, Piot P. Risk factors for mother-to-child transmission of human immunodeficiency virus-1 infection. Am J Obstet Gynecol. 1995;172(2 Pt 1):700-5.

26. Thorne C, Malyuta R, Semenenko I, Pilipenko T, Stelmah A, Posokhova S, et al. Mother-to-child transmission risk is increased among HIV-infected pregnant women in Ukraine with serological test results positive for syphilis. Clin Infect Dis. 2008;47(8):1114-5.

27. Van Dyke RB, Korber BT, Popek E, Macken C, Widmayer SM, Bardeguez A, et al. The Ariel Project: a prospective cohort study of maternal-child transmission of human immunodeficiency virus type 1 in the era of maternal antiretroviral therapy. The Journal of infectious diseases. 1999;179(2):319-28.

28. Yeganeh N, Watts HD, Camarca M, Soares G, Joao E, Pilotto JH, et al. Syphilis in HIV-infected mothers and infants: results from the NICHD/HPTN 040 study. The Pediatric infectious disease journal. 2015;34(3):e52-e7.

29. Bassett IV, Chetty S, Giddy J, Reddy S, Bishop K, Lu Z, et al. Screening for acute HIV infection in South Africa: finding acute and chronic disease. HIV medicine. 2011;12(1):46-53.

30. Jackson D, Naik R, Tabana H, Pillay M, Madurai S, Zembe W, et al. Quality of home‐based rapid HIV testing by community lay counsellors in a rural district of South Africa. Journal of the International AIDS Society. 2013;16(1):18744.

31. Kufa T, Kharsany AB, Cawood C, Khanyile D, Lewis L, Grobler A, et al. Misdiagnosis of HIV infection during a South African community‐based survey: implications for rapid HIV testing. Journal of the International AIDS Society. 2017;20:21753.

32. Wolpaw BJ, Mathews C, Chopra M, Hardie D, de Azevedo V, Jennings K, et al. The failure of routine rapid HIV testing: a case study of improving low sensitivity in the field. BMC health services research. 2010;10(1):1-4.

33. Lindbäck S, Thorstensson R, Karlsson AC, von Sydow M, Flamholc L, Blaxhult A, et al. Diagnosis of primary HIV-1 infection and duration of follow-up after HIV exposure. Aids. 2000;14(15):2333-9.

34. Johnson LF, Dorrington RE, Bradshaw D, Coetzee DJ. The role of sexually transmitted infections in the evolution of the South African HIV epidemic. Tropical Medicine & International Health. 2012;17(2):161-8.

35. Olson-Chen C, Balaram K, Hackney DN. Chlamydia trachomatis and adverse pregnancy outcomes: meta-analysis of patients with and without infection. Maternal and child health journal. 2018;22:812-21.

36. Vallely LM, Egli-Gany D, Wand H, Pomat WS, Homer CS, Guy R, et al. Adverse pregnancy and neonatal outcomes associated with Neisseria gonorrhoeae: systematic review and meta-analysis. Sexually transmitted infections. 2021;97(2):104-11.

37. Van Gerwen OT, Craig‐Kuhn MC, Jones AT, Schroeder JA, Deaver J, Buekens P, et al. Trichomoniasis and adverse birth outcomes: a systematic review and meta‐analysis. BJOG: An International Journal of Obstetrics & Gynaecology. 2021;128(12):1907-15.
